# Are invasive plants more competitive than native conspecifics? Patterns vary with competitors

**DOI:** 10.1038/srep15622

**Published:** 2015-10-22

**Authors:** Yulong Zheng, Yulong Feng, Alfonso Valiente-Banuet, Yangping Li, Zhiyong Liao, Jiaolin Zhang, Yajun Chen

**Affiliations:** 1 Key Laboratory of Tropical Forest Ecology, Xishuangbanna Tropical Botanical Garden, Chinese Academy of Sciences, Menglun, Mengla, Yunnan Province 666303, China; 2 College of Bioscience and Biotechnology, Shenyang Agricultural University, Shenyang, Liaoning Province 110866, China; 3 Departamento de Ecología de la Biodiversidad, Instituto de Ecología, Universidad Nacional Autónoma de México, Apartado Postal 70-275, C.P. 04510, México, D.F. México

## Abstract

Invasive plants are sometimes considered to be more competitive than their native conspecifics, according to the prediction that the invader reallocates resources from defense to growth due to liberation of natural enemies [‘Evolution of Increased Competitive Ability’ (EICA) hypothesis]. However, the differences in competitive ability may depend on the identity of competitors. In order to test the effects of competitors, *Ageratina adenophora* plants from both native and invasive ranges competed directly, and competed with native residents from both invasive (China) and native (Mexico) ranges respectively. Invasive *A*. *adenophora* plants were more competitive than their conspecifics from native populations when competing with natives from China (interspecific competition), but not when competing with natives from Mexico. Invasive *A. adenophora* plants also showed higher competitive ability when grown in high-density monoculture communities of plants from the same population (intrapopulation competition). In contrast, invasive *A. adenophora* plants showed lower competitive ability when competing with plants from native populations (intraspecific competition). Our results indicated that in the invasive range *A. adenophora* has evolved to effectively cope with co-occurring natives and high density environments, contributing to invasion success. Here, we showed the significant effects of competitors, which should be considered carefully when testing the EICA hypothesis.

It is frequently reported that invasive plants have a great impact on species composition, plant community structure, and ecosystem function, and that the invaders from invasive ranges are more competitive than their conspecifics from native ranges^1^,^2^. It has been proposed that once a plant is introduced into a new range, novel selection pressures from biotic and abiotic factors may induce evolutionary changes^3^,^4^. This may lead to ecologically important differentiation between plants from native and invasive populations. The Evolution of Increased Competitive Ability (EICA) hypothesis predicts that exotic plants may escape from the control of natural enemies in introduced ranges and gradually evolve to lose costly defense traits, reallocating resources and energy from defense to growth^5^. Siemann and Rogers^6^,^7^,^8^ found that *Sapium sebiferum* plants from invasive populations have higher competitive ability and lower leaf defensive ability than plants from native populations. Huang *et al.*^9^ also found that *Triadica sebifera* plants from invasive populations have higher biomass and lower defense to herbivores compared to plants from native populations.

The results for some invasive plants have also been inconsistent. Vilà *et al.*^10^ found that plants from invasive populations of *Hypericum perforatum* are not better competitors than plants from native populations. *Senecio pterophorus* plants grow similarly in the native and invasive ranges^11^, while the competitive ability of *Alliaria petiolata* plants from invasive populations is lower than that of plants from native populations^12^. Competitive conditions have significant impacts on the relative performance of plants from native and invasive ranges^13^. Leger and Rice^14^ found that introduced *Eschscholzia californica* from Chile is larger and more fecund than native Californian conspecifics only in the absence of competition. In contrast, Bossdorf *et al.*^12^ found that performance is similar for *Alliaria petiolata* plants from native and invasive populations in the absence of competition, whereas native plants outperform their invasive conspecifics when competing against each other. Increased or equal competitive ability has also been found for plants from invasive populations of other invaders (Table 1). The recent studies revealed that evolution indeed happened in invasive plants but little support for EICA hypothesis^15^,^16^.

The identity of competitors may influence the results of competitive experiments^17^. Native plants from the native range of an invasive plant may have adapted to the presence of the invader as they have a long co-evolutionary history; therefore, they may be less vulnerable to competition than natives from the invasive range of the invader. Under such circumstance, using native species from the native and invasive ranges of the invader as competitors may result in different conclusions regarding the intraspecific difference in the competitive ability of the invader. Similarly, the results of intraspecific competition may also differ with those of interspecific competition. Successful invasive plants often form dense monocultures in invasive ranges, whereas native conspecifics remain sparsely distributed in native ranges^18^. Thus, comparison with grown in monoculture, high density plantation might have less effects on invasive plants from the invasive ranges than native conspecifics.

In this study, we explored the effects of different competitors (natives from both ranges of the invader and the invader itself) on intraspecific differences in the competitive ability of the invasive *A. adenophora* using common garden experiments. *A. adenophora* is a perennial forb, native to Central America and Mexico, but a noxious invasive species in southern and southeastern Asia, eastern Australia, New Zealand, and southwestern Africa^19^. We studied performance differences between *A. adenophora* plants from native and invasive ranges in the absence of competitors (monoculture experiment), in the presence of intraspecific competitors (intraspecific competition experiment) and interspecific competitors from both ranges of the invader (interspecific competition experiment), and in an artificial monoculture community with high density (high density experiment). We also addressed the following questions: (1) Does *A. adenophora* gain or lose competitive ability against native species in its invasive range, (2) Does invasive *A. adenophora* also gain or lose intraspecific competitive ability, and (3) Does invasive *A. adenophora* also gain or lose competitive ability against native species in its original range?

## Results

**Figure 1:**
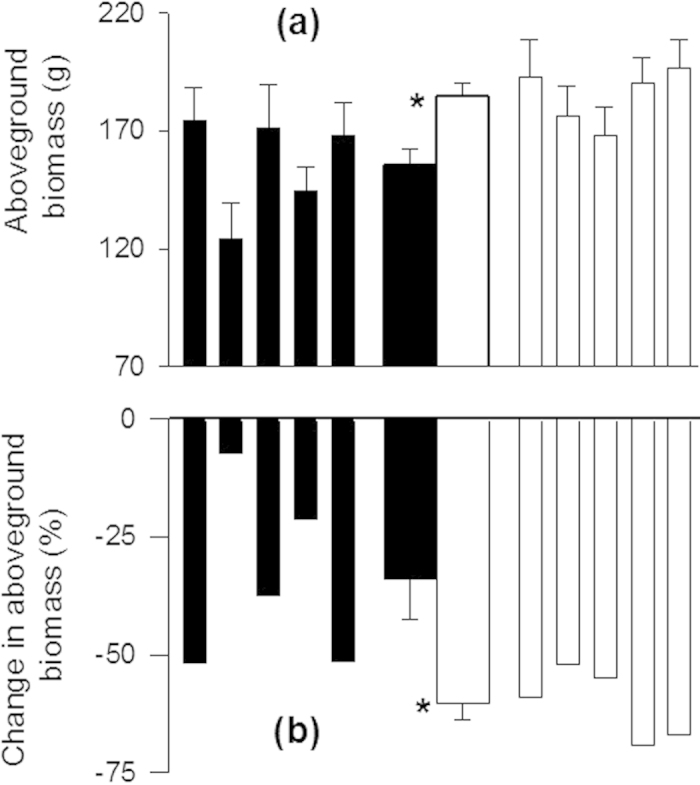
Aboveground biomass of *Ageratina adenophora* plants from the native (closed bars) and invasive (open bars) populations grown in monoculture (**a**), and changes in aboveground biomass caused by intraspecific competition (**b**). Narrow bars indicate means and SE for each population; two thicker bars in the center depict means and SE for each range. * indicates significant differences between ranges (*P* < 0.05) in aboveground biomass (one-way nested ANOVAs) and percentage change in aboveground biomass (*t*-test).

The identity of competitors had a significant effect on the competitive ability of native and invasive *A. adenophora* plants. When grown in monoculture, total biomass was not significantly different between *A. adenophora* plants from native and invasive ranges (Appendix 1a), although the plants from invasive populations produced more aboveground biomass (Fig. 1a; Table 2). The *A. adenophora* plants from invasive populations had lower root biomass fraction than those from the native range (Appendix 1b). When invasive *A. adenophora* plants competed with their native conspecifics (intraspecific competition), the decrease in aboveground biomass was significantly higher for the plants from invasive populations (Fig. 1b; Table 2).

**Figure 2:**
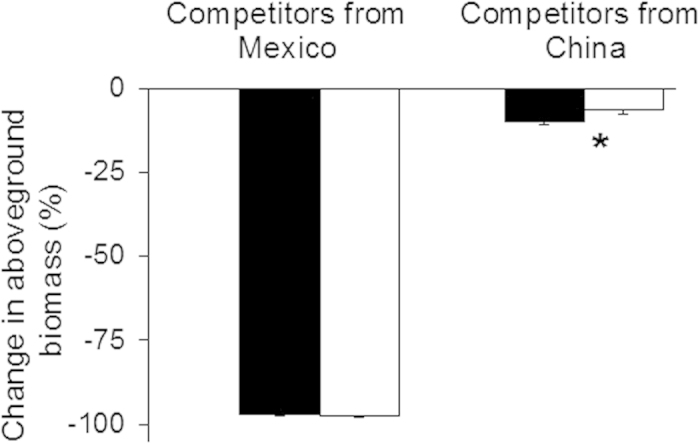
Changes in aboveground biomass of *Ageratina adenophora* plants from the native (closed bars) and invasive (open bars) populations caused by competition of two resident native species from Mexico (native range) and China (invasive range), respectively. * indicates significant differences between ranges (*P* < 0.05) (one-way nested ANOVAs).

When grown in monoculture, the aboveground biomass of the two native species from the invasive range of the invader (China) was significantly lower than that of *A*. *adenophora* plants from both native and invasive ranges (Appendix 2a). However, the differences in aboveground biomass were not significantly different between the two native species from the native range of the invader (Mexico) and *A*. *adenophora* from both ranges (Appendix 3a). Competition from the natives of both ranges (interspecific competition) decreased the aboveground biomass of *A*. *adenophora* from both ranges, and the decrease was significantly higher when the natives from Mexico were used as competitors (Fig. 2; Table 2). However, the differences in competitive ability were not significant between *A*. *adenophora* plants from native and invasive populations when competing with the two natives from Mexico (Fig. 2; Table 2; Appendix 3a). When they competed with the natives from China, *A*. *adenophora* plants from invasive populations had significantly higher competitive ability than those from native populations (Fig. 2; Appendix. 2b). Compared to *A*. *adenophora* from both ranges, the interspecific competitive ability was lower for the natives from China, but higher for the natives from Mexico (Appendix 2b, 3b).

**Figure 3:**
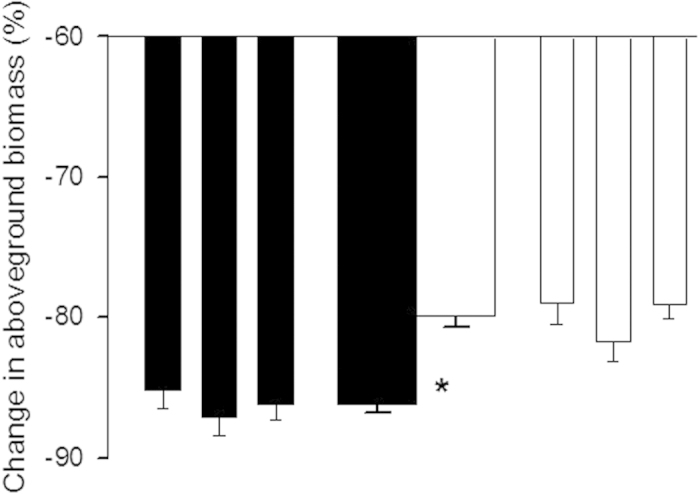
Changes in aboveground biomass of *Ageratina adenophora* plants from the native (closed bars) and invasive (open bars) populations when grown in artificial communities with high density. Narrow bars indicate means and SE for each population; two thicker bars in the center depict means and SE for each range. * indicates significant differences between ranges (*P* < 0.05) (one-way nested ANOVAs).

When grown at high individual density (intrapopulation competition), the decrease in aboveground biomass was higher for *A*. *adenophora* plants from native populations than those from invasive populations (Fig. 3; Table 2).

## Discussion

Our results indicated that the differences in competitive ability between *A. adenophora* plants from native and invasive populations were inconsistent, depending on the competitors used in our common garden experiment. Thus, it is important to take into account the effects of competitors when testing the EICA (Evolution Increased Competitive Ability) hypothesis, choose suitable competitors according to the specific purpose, and explain experimental results carefully. In addition, abiotic environments also influence experimental results^13^.

Higher aboveground biomass did not lead to higher intraspecific competitive ability for *A*. *adenophora* plants from the invasive range, results that were inconsistent with the prediction of the EICA hypothesis^5^,^17^,^18^. The lower intraspecific competitive ability of *A*. *adenophora* plants from the invasive range may be associated with their lower root mass fraction (Appendix 1b). Several studies have also found that the competitive advantages of invasive species are associated with shifts in biomass allocation rather than increased individual size^20^,^21^,^22^,^23^. The decreased biomass allocation to roots in invasive populations of *A*. *adenophora* may be associated with improved soil environments (moisture, nutrients, or microbes) in the invasive range. Annual precipitation was significantly higher in the invasive range than in the native range (Appendix 4). Plants generally decrease biomass allocation to roots in benign belowground conditions^24^,^25^. Reallocation of biomass from roots to aboveground parts allows invasive *A*. *adenophora* to be more effective in competing for light. Natural selection may favor genotypes with increased light capture ability in invasive ranges with increased precipitation, contributing to successful competition with native species. However, the lower root mass fraction may be a disadvantage of *A*. *adenophora* in native ranges with lower precipitation. Thus, in our study site located in Tlayacapan, Mexico, *A*. *adenophora* plants from the native range with higher root mass fraction showed higher competitive ability than those from the invasive range with lower root mass fraction.

The higher interspecific competitive ability of *A*. *adenophora* plants from the invasive range when they competed with the natives from the invasive range (China) of the invader may be associated with their greater aboveground biomass and stronger allelopathic effects compared to those from the native range. The natives from China grew more slowly than *A*. *adenophora* plants from both ranges (Appendix 2). When competing with the slowly growing natives from China, the higher biomass allocation to shoot might provide a competitive advantage to the invasive *A*. *adenophora* compared to its native conspecifics^20^. It has been reported that the invader may use dense canopy to outshade competitors in the invasive range^26^. Previous studies have demonstrated that *A*. *adenophora* has strong allelopathic effects on neighboring plants^27^,^28^. Consistently with the novel weapons hypothesis^17^,^18^, native species from the invasive range of *A*. *adenophora* were more vulnerable to the allelochemicals of the invader than the natives from the native range of the invader^29^. In this case, natural selection may favor the genotypes with increased allelopathic effects in the invasive range of *A. adenophora*. Concentrations of some allelochemicals were indeed higher in *A. adenophora* plants from the invasive range than in those from the native range^29^. The higher allelopathic effect of *A. adenophora* plants from the invasive range may contribute to higher competitive ability when competing with natives from China, which are vulnerable to the allelochemicals of the invader. Increased allelochemical-driven competitive advantage was also found in other invasive plants^18^.

However, the greater aboveground biomass and stronger allelopathic effects of *A*. *adenophora* plants from the invasive range did not lead to higher interspecific competitive ability when they competed with the natives (*Cosmos sulphureus* and *Aldama dentata*) from the native range (Mexico) of the invader.It might due to *C. sulphureus* and *A. dentata* have long co-evolutionar history with *A. adenophora*, they might not be sensitive to the allelochemicals of *A. adenophora*. So, stronger allelopathic effects of *A. adenophora* plants from the invasive range did not contribute to higher competitive ability. In addition, *C. sulphureus* and *A. dentata* grew much faster than *A. adenophora* in the early period of the interspecific competition experiment, although the final aboveground biomass was similar among species. The biomass of the natives from Mexico almost reached its final values in 3 months (personal observation). The faster growth rates of the natives from Mexico not only contributed to their higher competitive ability compared to *A. adenophora* from both ranges (Appendix 3b), but also eliminated the competitive advantage of *A. adenophora* plants from the invasive range. The increased aboveground biomass did not increase the competitive ability of *A. adenophora* plants from the invasive range as they grew under the canopy of the natives from Mexico, and could not effectively shade the competitors.In addition, the stronger allelopathic effects of *A. adenophora* plants from the invasive range might not increase their competitive ability when competing with natives from Mexico, which might not vulnerable to allelochemicals of the invader as they share long co-evolutionary history.

In the native range, *A. adenophora* plants are often sparsely distributed, whereas they generally form dense monocultures in the invasive range (personal observation). *A. adenophora* may have evolved certain adaptive strategies (morphological or physiological) in the invasive range to cope with high-density environments. Therefore, the decrease in aboveground biomass was less in *A. adenophora* plants from the invasive range than in those from the native range when grown in high-density (Fig. 3).

Many factors can cause evolutionary changes in invasive plants, for example novel assemblages of enemies and plants and new abiotic environments in introduced ranges^5^,^12^,^30^,^31^. Novel biotic and abiotic environments may lead to new competitive strategies for invasive plants, which may be quite different from those that they have in their native ranges. The differences in competitive ability between plants from native and invasive ranges may be different when different competitors are used (Figs 1, 2, 3) or under different abiotic conditions^13^.

In conclusion, *A. adenophora* plants from the invasive range showed higher interspecific competitive ability than those from the native range when competing with native species from the invasive range of the invader, but not when competing with native species from the native range of the invader. Plants from the invasive range of the invader also showed higher intrapopulation competitive ability when grown in high density. The increased ability to deal with co-occurring natives and strongly competitive environments may contribute to the success of the invader in the invasive range. In contrast, *A. adenophora* plants from the invasive range showed lower intraspecific competitive ability. Our results indicated that the differences in competitive ability between plants from native and invasive populations are competitor-dependent, which should be considered when testing the EICA hypothesis.

## Methods

### Study sites and materials

This study was conducted within the native range of *A. adenophora* in Tlayacapan, Morelos, Mexico (18°57′N, 98°58′W; 1634 m above sea level). The mean annual temperature of this area is 19.3 °C, the mean temperature of the hottest month (June) is 22.9 °C, and the mean temperature of the coolest month (January) is 16.9 °C. The mean annual precipitation is 988 mm with a dry period from November to April^32^.

In 2006, we collected seeds of *A. adenophora* from five populations in the native range (Mexico) and five populations in the invasive range (three in China and two in India) (Appendix 5). Seeds were collected from more than 10 individuals that were at least 20 m apart from one another. In order to exclude maternal effect, we used seeds of the next generation. Seeds were germinated in a seedbed in December 2006. In February 2007, when the seedlings were approximately 10 cm tall, 200 similarly sized seedlings (20 per population) were planted in a common garden located in Menglun, Mengla County, Yunnan Province, southwest China (21°56′N, 101°150′E; 570 m above sea level). The reproduction system of *A. adenophora* is apomixis^33^, which avoids hybridization among different populations. In May 2008, *A. adenophora* seeds of each population were collected.

In 2009, we collected seeds from the native *Cosmos sulphureus* and *Aldama dentata* in the native range of *A. adenophora* (Mexico) and from the native *Eupatorium japonicum* and *E. stoechadosmum* in the invasive range of *A. adenophora* (China). All these species were sympatric and ecologically similar to *A. adenophora*. For each of these species, seeds were also collected from more than 10 individuals.

### Common garden experiments

In April 2010, seeds of *A*. *adenophora* from each population, and the four natives from Mexico and China were sown separately into a seed bed in a greenhouse in Tlayacapan. In June 2010, similar-sized vigorous seedlings (approximately 10 cm tall) were transplanted into a common garden, in which we established 69 rectangular plots (6.5 m × 60 cm) and 18 square plots (1 m × 1 m). The seedlings of *A*. *adenophora* were grown under four conditions: monoculture, intraspecific competition, interspecific competition, and high density (Appendix 6).

For monoculture, we used 10 rectangular plots and in each of those we grew one seedling of *A*. *adenophora* from each population (10 replicates). Four rectangular plots were used for monoculture of seedlings of four native species. Due to the limited number of seedlings, for intraspecific competition, we grew seedlings of *A*. *adenophora* from each native population with one seedling from the three randomly selected invasive populations (6 cm apart between competitors). There were three competition combinations for each population and 15 combinations in total. Each rectangular plot contained 10 different competing pairs, and there were 10 replicates for each competing pair (150 competing pairs in total grown in 15 rectangular plots). For interspecific competition, we grew one seedling of *A*. *adenophora* from each population with one seedling from each of the four native species. There were also 10 replicates for each competing pair (400 competing pairs in total grown in 40 rectangular plots). Individual seedlings or competing pairs were planted 60 cm apart from any other seedling or seedling pair. The 65 rectangular plots were randomly distributed in the garden (Appendix 6). Due to the limited number of seedlings for several populations of *A*. *adenophora*, seedlings of *A*. *adenophora* from only six populations (three invasive and three native) were used in the artificial monoculture community with high density. Forty-nine seedlings from each of the six populations were transplanted into a square plot (1 m × 1 m), and there were three replicates for each population (18 square plots). These 18 square plots, which were randomly distributed in the garden (Appendix 6), were used to mimic communities that are dominated by *A*. *adenophora* at high density.

In February 2011, the aboveground parts of all plants were harvested, oven-dried at 60 °C for 48 h, and weighed. In order to avoid border effect in the high-density growth experiment, 10 *A*. *adenophora* seedlings were randomly harvested in the center part of each plot. In order to test whether biomass reallocation occurred after introduction, roots of *A*. *adenophora* plants from both ranges grown in monoculture were collected, washed, oven-dried at 60 °C for 48 h, and weighed. Root mass fraction was calculated as the ratio of root mass to total mass. Roots were not collected for plants grown in under intraspecific and interspecific competition and high density because the roots of two or more individuals often twined together, increasing the difficulty to separate the roots of different individual.

Response to competition was measured as the percentage change in aboveground biomass, i.e., [(Biomass_comp_ − Biomass_mono_)/Biomass_mono_] × 100%, where Biomass_mono_ is the mean aboveground biomass of *A*. *adenophora* plants from each population or each of the four native species grown in monoculture, and Biomass_comp_ is the mean aboveground biomass of *A*. *adenophora* plants from each population or each of the four native species when grown with competitors.

### Statistical analysis

The differences in total biomass, aboveground biomass, and root mass fraction between *A*. *adenophora* plants from native and invasive ranges grown in monoculture were tested using one-way nested ANOVA (Fig. 1a; Appendix 1). Range was used as a fixed factor, and population nested within range was used as a random factor. The differences in the percentage change of aboveground biomass caused by intraspecific competition between *A*. *adenophora* plants from native and invasive ranges were compared using *t*-tests (Fig. 1b). The differences in the percentage changes of aboveground biomass between *A*. *adenophora* plants from native and invasive ranges grown in high density were tested using one-way nested ANOVA (Fig. 3). Range was used as a fixed factor, and population nested within range was used as a random factor. The difference in aboveground biomass and the percentage change of biomass between native and invasive populations of *A*. *adenophora* species native to China or Mexico was determined using one-way ANOVA (Fig. 2; Appendix 2, 3). The differences in annual precipitation between ranges were tested using one-way ANOVA (Appendix 6).

## Additional Information

**How to cite this article**: Zheng, Y. *et al.* Are invasive plants more competitive than native conspecifics? Patterns vary with competitors. *Sci. Rep.* **5**, 15622; doi: 10.1038/srep15622 (2015).

## Supplementary Material

Supporting Information

## Figures and Tables

**Table 1:** Differences in competitive ability between plants originated from invasive and native ranges of eight species reported in references.

Species	Competitive ability	Competitors	References
*Eschscholzia californica*	No	IN	Leger and Rice (2003)^14^
*Hypericum perforatum*	No	IN	Vilà *et al.* (2003)^10^
*Alliaria petiolata*	Decrease	D	Bossdorf *et al.* (2004)^12^
*Silene latifolia*	No	INV	Blair and Wolfe (2004)^34^
*Centaurea maculosa*	Increase	IV	Ridenour *et al.* (2008)^35^
*Dactylis glomerata*	Decrease	D	Liesfo *et al.* (2012)^36^
*Solidago canadensis*	Increase	IV	Yuan *et al.* (2013)^31^
*Chromolaena odorata*	Increase	D	Zheng *et al.* (2015)^18^

“Increase” indicates higher competitive ability for plants from invasive populations compared with plants from native populaitons; “Decrease” indicates lower competitive ability for plants from invasive populations; “No” indicates similar competitive ability between plants from invasive and native populations. “IN” indicates that the competitors are resident native species from native range; “IV” indicates that the competitors are resident native species from invasive range; “INV” indicates that the competitors are resident native species from both ranges; “D” indicates that plants from invasive populations were competed with plants from native populations.

**Table 2:** Differences in aboveground biomass when grown in monoculture between native and invasive ranges, and differences in change in aboveground biomass between native and invasive ranges when grown in intraspecific competition, interspecific competition and high density plantation according to one-way nested ANOVAs analysis.

Experiment	Variable	df	F-value	P
**Monoculture**	Aboveground biomass	1	6.555	0.034
**Intraspecific competition**	Change in Aboveground biomass	1	10.183	0.013
Interspecific competitionv				
with native species from Mexico	Change in Aboveground biomass	1	0.413	0.528
with native species from China	Change in Aboveground biomass	1	5.629	0.029
**High density plantation**	Change in Aboveground biomass	1	35.4	<0.001

## References

[b1] D’Antonio C. M. & Kark S. Impacts and extent of biotic invasions in terrestrial ecosystems. Trends Ecol. Evol. 17, 202–204 (2002).

[b2] Rout M. & Callaway R. M. An invasive plant paradox. Science 324, 734–735 (2009). 19423809 10.1126/science.1173651

[b3] Mooney H. A. & Cleland E. E. The evolutionary impact of invasive species. Proc. Natl. Acad. Sci. USA 98, 5446–5451 (2001). 11344292 10.1073/pnas.091093398 PMC33232

[b4] Hufbauer R. A. *et al.* Anthropogenically-induced adaptation to invade (AIAI: contemporary adaptation to human-altered habitats within the native range can promote invasions). Evol. Appl. 5, 89–101 (2012). 25568032 10.1111/j.1752-4571.2011.00211.x PMC3353334

[b5] Blossey B. & Nötzold R. Evolution of increased competitive ability in invasive non-indigenous plants: a hypothesis. J. Ecol. 83, 887–889 (1995).

[b6] Siemann E. & Rogers W. E. Genetic differences in growth of an invasive tree species. Ecol. Lett. 4, 514–518 (2001).

[b7] Siemann E. & Rogers W. E. Increased competitive ability of an invasive tree limited by an invasive beetle. Ecol. Appl. 13, 1503–1507 (2003a).

[b8] Siemann E. & Rogers W. E. Reduced resistance of invasive varieties of the alien tree *Sapium sebiferum* to a generalist herbivore. Oecologia, 135, 451–457 (2003b). 12721836 10.1007/s00442-003-1217-4

[b9] Huang W., Carrillo J., Ding J. Q. & Siemann E. Invader partitions ecological and evolutionary responses to above- and belowground herbivory. Ecology, 93, 2343–2352 (2012). 23236906 10.1890/11-1964.1

[b10] Vilà M., Gómez A. & Maron J. L. Are alien plants more competitive than their native conspecifics? A test using *Hypericum perforatum* L. Oecologia 137, 211–215 (2003). 12883989 10.1007/s00442-003-1342-0

[b11] Caño L., Escarré J., Vrieling K. & Sans F. X. Palatability to a generalist herbivore, defence and growth of invasive and native *Senecio* species: testing the evolution of increased competitive ability hypothesis. Oecologia 159, 95–106 (2009). 18941785 10.1007/s00442-008-1182-z

[b12] Bossdorf O., Prati D., Auge H. & Schmid B. Reduced competitive ability in an invasive plant. Ecol. Lett. 7, 346–353 (2004).

[b13] Liao Z. Y., Zhang R., Barclay G. F. & Feng Y. L. Differences in competitive ability between plants from nonnative and native populations of a tropical invader relates to adaptive responses in abiotic and biotic environments. PLoS ONE, 8, e71767 (2013). 23977140 10.1371/journal.pone.0071767 PMC3745391

[b14] Leger E. A. & Rice K. J. Invasive California poppies (*Eschscholzia californica* Cham.) grow larger than native individuals under reduced competition. Ecol. Lett. 6, 257–264 (2003).

[b15] Felker-Quinn E., Schweitzer J. A. & Bailey J. K. Meta-analysis reveals evolution in invasive plant species but little support for Evolution of Increased Competitive Ability (EICA). Ecol. Evol. 3, 739–751 (2013). 23531703 10.1002/ece3.488 PMC3605860

[b16] Lowry E. *et al.* Biological invasions: a field synopsis, systematic review, and database of the literature. Ecol. Evol. 3, 182–196 (2013). 23404636 10.1002/ece3.431 PMC3568853

[b17] Qin R. M. *et al.* The evolution of increased competitive ability, innate competitive advantages, and novel biochemical weapons act in concert for a tropical invader. New Phytol. 197, 979–988 (2013). 23252450 10.1111/nph.12071

[b18] Zheng Y. L. *et al.* Integrating novel chemical weapons and evolutionarily increased competitive ability in success of a tropical invader. New phytol. 205, 1350–1359 (2015) 25367824 10.1111/nph.13135

[b19] Cronk Q. C. B. & Fuller J. L. Plant invaders: the threat to natural ecosystems (1995).

[b20] te Beest M., Stevens N., Olff H. & van der Putten W. H. Plant-soil feedback induces shifts in biomass allocation in the invasive plant *Chromolaena odorata*. J. Ecol. 97, 1281–1290 (2009).

[b21] Pattison R. R., Goldstein G. & Ares A. Growth, biomass allocation and photosynthesis of invasive and native Hawaiian rainforest species. Oecologia 117, 449–459 (1998). 10.1007/s004420050680 28307669

[b22] Morrison J. A. & Mauck K. Experimental field comparison of native and non-native maple seedlings: natural enemies, ecophysiology, growth and survival. J. Ecol. 95, 1036–1049 (2007).

[b23] Meyer G. & Hull-Sanders H. Altered patterns of growth, physiology and reproduction in invasive genotypes of *Solidago gigantea* (Asteraceae). Biol. Invasions 10, 303–317 (2008).

[b24] Iwasa Y. & Roughgarden J. Shoot ⁄ root balance of plants: optimal growth of a system with many vegetative organs. Theor. Popul. Biol. 25, 78–105 (1984).

[b25] Poorter H. *et al.* Biomass allocation to leaves, stems and roots: meta-analyses of interspecific variation and environmental control. New phytol. 193, 30–50 (2012). 22085245 10.1111/j.1469-8137.2011.03952.x

[b26] Feng Y. L., Wang J. F. & Sang W. G. Biomass allocation, morphology and photosynthesis of invasive and noninvasive exotic species grown at four irradiance levels. Acta Oecol. 31, 40–47 (2007).

[b27] Zheng L. & Feng Y. L. Allelopathic effects of *Eupatorium adenophorum* Spreng. on seed germination and seedling growth in ten herbaceous species. Acta Ecol. Sin. 25, 2782–2787 (2005).

[b28] Han L. H. & Feng Y. L. The effects of growth and development stage on ailelopathy of *Eupatorium adenophorum*. Acta Ecol. Sin. 27, 1185–1191 (2007).

[b29] Inderjit, *et al.* Volatile chemicals from leaf litter are associated with invasiveness of a neotropical weed in Asia. Ecology 92, 316–324 (2011). 21618911 10.1890/10-0400.1

[b30] He W. M., Thelen G. C., Ridenour W. M. & Callaway R. M. Is there a risk to living large? Large size correlates with reduced growth when stressed for knapweed populations. Biol. Invasions 12, 3591–3598 (2010).

[b31] Yuan Y. G. *et al.* Enhanced allelopathy and competitive ability of invasive plant *Solidago canadensis* in its introduced range. *J*. Plant Ecol. 6, 253–263 (2013).

[b32] García E. in Modificaciones al sistema de clasificación cimática de Köppen, para adaptarlo a las condiciones de la República Mexicana Cuarta edición (ed. García E.) 220 (UNAM, 1988).

[b33] Baker H. G. [The modes of origin of weeds]. The Genetics of Colonizing Species [Baker H. G. & Stebbines G. L. (eds.)]. 147–168 (Academic Press, New York, 1965).

[b34] Blair A. C. & Wolfe L. M. The evolution of an invasive plant: an experimental study with *Silene latifolia*. Ecology 85, 3035–3042 (2004).

[b35] Ridenour W. M., Vivanco J. M., Feng Y. L., Horiuchi J. & Callaway R. M. No evidence for tradeoffs: Centaurea plants from America are better competitors and defenders than plants from the native range. Ecol. Monogr. 78, 369–386 (2008).

[b36] Leifso A. *et al.* Expansion of a globally pervasive grass occurs without substantial trait differences between home and away populations. Oecologia 170, 1123–1132 (2012). 22669263 10.1007/s00442-012-2370-4

