# Supplementary material for: Are invasive plants more competitive than native conspecifics? Patterns vary with competitors
Source: Sci Rep. 2015 Oct 22;5:15622. doi: 10.1038/srep15622 (PMC4650833; doi:10.1038/srep15622)
Supplement: Supporting Information [file srep15622-s1.pdf]

Are invasive plants more competitive than native conspecifics? Patterns vary with competitors

Yulong Zheng[1,*], Yulong Feng[2,*], Alfonso Valiente-Banuet[3], Yangping Li[1], Zhiyong Liao[1], Jiaolin Zhang[1], Yajun Chen[1]

[1]*Key Laboratory of Tropical Forest Ecology, Xishuangbanna Tropical Botanical Garden, Chinese Academy of Sciences, Menglun, Mengla, Yunnan Province 666303, China*

[2]*College of Bioscience and Biotechnology, Shenyang Agricultural University, Shenyang, Liaoning Province 110866, China*

[3]*Departamento de Ecología de la Biodiversidad, Instituto de Ecología, Universidad Nacional Autónoma de México, Apartado Postal 70-275, C.P. 04510, México, D.F. México*

[*]Corresponding authors: zhengyl@xtbg.org.cn; fyl@xtbg.ac.cn

Supporting information

Appendix 1. Total biomass (a) and root biomass fraction (b) for *Ageratina adenophora* plants from the native (closed bars) and invasive (open bars) populations grown in monoculture. Narrow bars indicate the means and SE for each population; two thicker bars in the center depict means and SE for each range. * indicates significant differences ($P < 0.05$) between ranges (one-way nested ANOVAs).

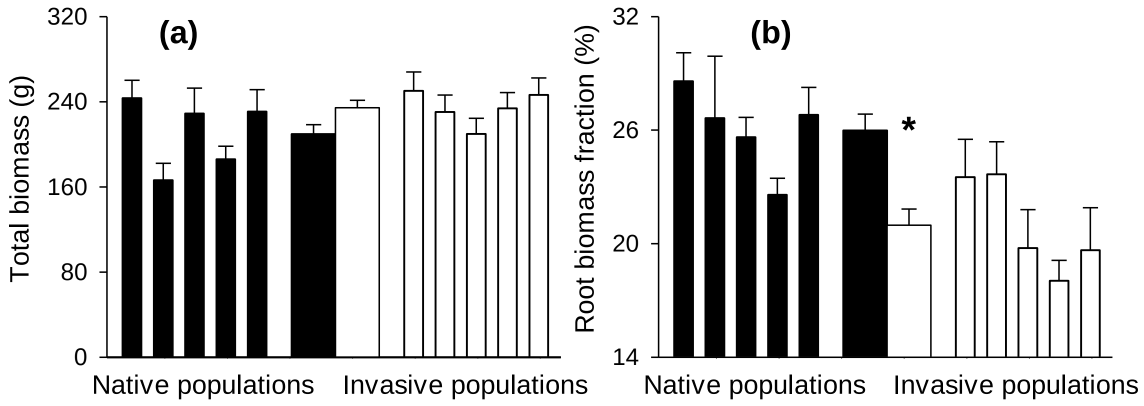

Appendix 2. Aboveground biomass for plants grown in monoculture (a) and change in this variable caused by competition (b) for native (closed bars) and invasive (open bars) populations of *Ageratina adenophora*, *Eupatorium stoechadosmum* (criss-cross bars; native to China), and *E. japonicum* (horizontally striped bars; native to China). Changes in the aboveground biomasses of both native and invasive populations of *A. adenophora* were caused by both native species. For each native species, changes in the aboveground biomass were caused by both native and invasive populations of *A. adenophora*. Mean and SE are shown. Different letters indicate significant differences among species at $P < 0.05$ according to one-way ANOVAs.

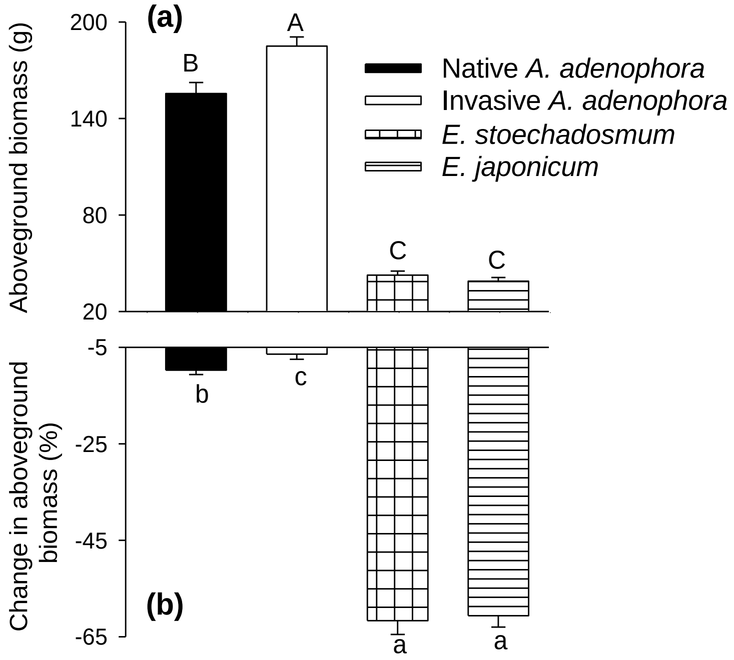

Appendix 3. Aboveground biomass of plants grown in monoculture (a) and changes in this variable caused by competition (b) for native (closed bars) and invasive (open bars) populations of *Ageratina adenophora*, *Cosmos sulphureus* (diagonal bars; native to Mexico), and *Aldama dentata* (cross-hatched bars; native to Mexico). Changes in the aboveground biomasses of both native and invasive populations of *A. adenophora* were caused by both native species. For each native species, changes in the aboveground biomass were caused by both native and invasive populations of *A. adenophora*. Mean and SE are shown. Different letters indicate significant differences among species at $P < 0.05$ according to one-way ANOVAs.

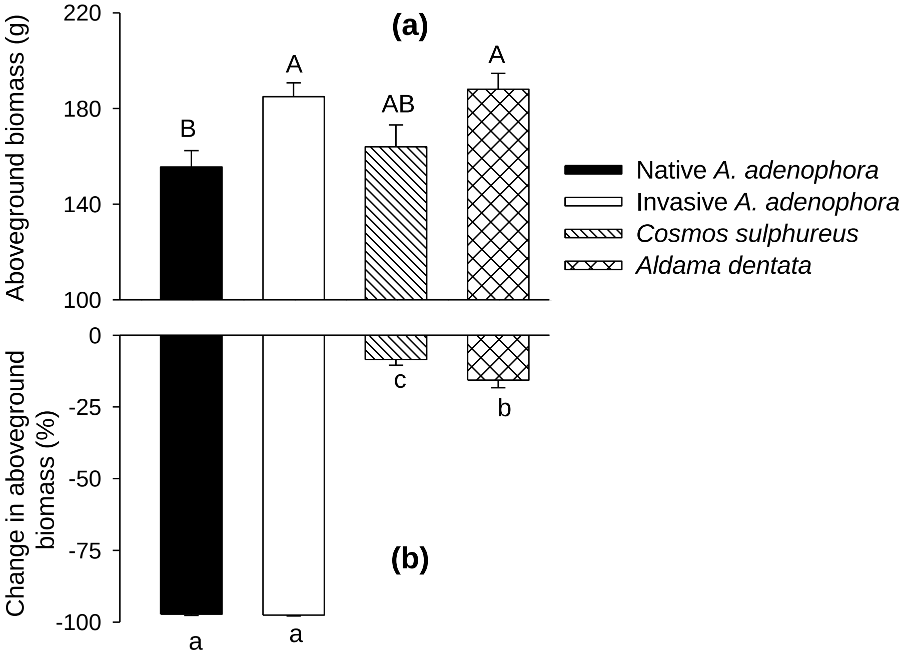

Appendix 4. Annual precipitation for each site where the seeds of *Ageratina adenophora* were collected. Narrow bars indicate annual precipitation for each site; two thicker bars in the center depict means and SE for each range. *** indicates significant differences ($P < 0.01$) between ranges (*t*-test).

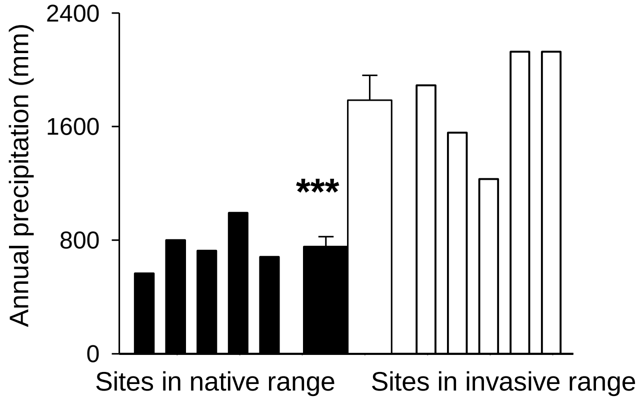

Appendix 5. Background information on sampled populations of *Ageratina adenophora* and native species from China and Mexico used in this study

The information on annual precipitation of each site in Mexico is from Mexican National Commission of Water "Comisión Nacional del Agua" (http://conagua.gob.mx/inicio.aspx), and the information on each site in China is from meteorologic bureau of each county.

Species	Site	Latitude	Longitude	Annual precipitation (mm)
Invasive *A. adenophora*	Zhutang, Lancang, Yunnan, China	22°45'N	99°45'E	1890
	Menglun, Mengla, Yunnan, China	21°57'N	101°12'E	1557
	Zhenyuan, Yunan, China	24°01'N	101°05'E	1230
	Mussoorie, Uttrakhand, India	30°25'N	78°04'E	2127
	Mussoorie, Uttrakhand, India	30°26'N	78°44'E	2127
Native *A. adenophora*	San Miguel de Allende Guanajuato, Mexico	20°55'N	100°45'W	566.1
	Umecuaro, Michoacán, Mexico	19°42'N	101°11'W	799.7
	Acebuche, Tarimoro, Guanajuato, Mexico	20°25'N	102°10'W	725.4
	Pinal de Amoles, Querétaro, Mexico	20°35'N	100°23'W	992
	Querendaro, Michoacán, Mexico	19°52'N	100°54'W	682.3
Eupatorium stoechadosmum	Menglun, Mengla, Yunnan, China	21°56'N	101°15'E	1557
E. japonicum	Kunming, Yunnan, Chian	25°05'N	102°49'E	1035
Cosmos sulphureus	Tlayacapan, Morelos, Mexico	18°57'N	98°58'E	988
Aldama dentata	Tlayacapan, Morelos, Mexico	18°57'N	98°58'W	988

Appendix 6. The distribution of each experimental plot in the common garden. I_1-I_5 indicate *Ageratina adenophora* plants from the five invasive populations, respectively; N_1-N_5 indicate *A. adenophora* plants from the five native populations, respetively. A and B indicate the two native species from China (*Eupatorium stoechadosmum* and *E. japonicum*, respectively); C and D indicate the two native species from Mexico (*Cosmos sulphureus* and *Aldama dentata*, respectively).

I_1	N_1	I_2	N_2	I_3	N_3	I_4	N_4	I_5	N_5
I_1N_1	I_2N_2	I_3N_3	I_4N_4	I_5N_5	I_1N_4	I_2N_5	I_3N_1	I_4N_2	I_5N_3
I_1N_3	I_2N_4	I_3N_5	I_4N_1	I_5N_2	I_1N_1	I_2N_2	I_3N_3	I_4N_4	I_5N_5
I_1A	N_1A	I_2A	N_2A	I_3A	N_3A	I_4A	N_4A	I_5A	N_5A
I_1B	N_1B	I_2B	N_2B	I_3B	N_3B	I_4B	N_4B	I_5B	N_5B
I_1C	N_1C	I_2C	N_2C	I_3C	N_3C	I_4C	N_4C	I_5C	N_5C
I_1D	N_1D	I_2D	N_2D	I_3D	N_3D	I_4D	N_4D	I_5D	N_5D
I_2 I_2 I_2 I_2 I_2 I_2 I_2<br>I_2 I_2 I_2 I_2 I_2 I_2 I_2<br>I_2 I_2 I_2 I_2 I_2 I_2 I_2<br>I_2 I_2 I_2 I_2 I_2 I_2 I_2<br>I_2 I_2 I_2 I_2 I_2 I_2 I_2<br>I_2 I_2 I_2 I_2 I_2 I_2 I_2				N_1 N_1 N_1 N_1 N_1 N_1 N_1<br>N_1 N_1 N_1 N_1 N_1 N_1 N_1<br>N_1 N_1 N_1 N_1 N_1 N_1 N_1<br>N_1 N_1 N_1 N_1 N_1 N_1 N_1<br>N_1 N_1 N_1 N_1 N_1 N_1 N_1<br>N_1 N_1 N_1 N_1 N_1 N_1 N_1<br>N_1 N_1 N_1 N_1 N_1 N_1 N_1				I_3 I_3 I_3 I_3 I_3 I_3 I_3<br>I_3 I_3 I_3 I_3 I_3 I_3 I_3<br>I_3 I_3 I_3 I_3 I_3 I_3 I_3<br>I_3 I_3 I_3 I_3 I_3 I_3 I_3<br>I_3 I_3 I_3 I_3 I_3 I_3 I_3<br>I_3 I_3 I_3 I_3 I_3 I_3 I_3<br>I_3 I_3 I_3 I_3 I_3 I_3 I_3	
I_1	N_1	I_2	N_2	I_3	N_3	I_4	N_4	I_5	N_5
I_1N_4	I_2N_5	I_3N_1	I_4N_2	I_5N_3	I_1N_3	I_2N_4	I_3N_5	I_4N_1	I_5N_2
I_1N_1	I_2N_2	I_3N_3	I_4N_4	I_5N_5	I_1N_4	I_2N_5	I_3N_1	I_4N_2	I_5N_3
I_1A	N_1A	I_2A	N_2A	I_3A	N_3A	I_4A	N_4A	I_5A	N_5A
I_1B	N_1B	I_2B	N_2B	I_3B	N_3B	I_4B	N_4B	I_5B	N_5B
I_1C	N_1C	I_2C	N_2C	I_3C	N_3C	I_4C	N_4C	I_5C	N_5C
I_1D	N_1D	I_2D	N_2D	I_3D	N_3D	I_4D	N_4D	I_5D	N_5D
A	B	C	D	A	B	C	D	A	B
N_3 N_3 N_3 N_3 N_3 N_3 N_3<br>N_3 N_3 N_3 N_3 N_3 N_3 N_3<br>N_3 N_3 N_3 N_3 N_3 N_3 N_3<br>N_3 N_3 N_3 N_3 N_3 N_3 N_3<br>N_3 N_3 N_3 N_3 N_3 N_3 N_3<br>N_3 N_3 N_3 N_3 N_3 N_3 N_3<br>N_3 N_3 N_3 N_3 N_3 N_3 N_3				I_4 I_4 I_4 I_4 I_4 I_4 I_4<br>I_4 I_4 I_4 I_4 I_4 I_4 I_4<br>I_4 I_4 I_4 I_4 I_4 I_4 I_4<br>I_4 I_4 I_4 I_4 I_4 I_4 I_4<br>I_4 I_4 I_4 I_4 I_4 I_4 I_4<br>I_4 I_4 I_4 I_4 I_4 I_4 I_4<br>I_4 I_4 I_4 I_4 I_4 I_4 I_4				N_5 N_5 N_5 N_5 N_5 N_5 N_5<br>N_5 N_5 N_5 N_5 N_5 N_5 N_5<br>N_5 N_5 N_5 N_5 N_5 N_5 N_5<br>N_5 N_5 N_5 N_5 N_5 N_5 N_5<br>N_5 N_5 N_5 N_5 N_5 N_5 N_5<br>N_5 N_5 N_5 N_5 N_5 N_5 N_5<br>N_5 N_5 N_5 N_5 N_5 N_5 N_5	
I_1	N_1	I_2	N_2	I_3	N_3	I_4	N_4	I_5	N_5
I_1N_3	I_2N_4	I_3N_5	I_4N_1	I_5N_2	I_1N_1	I_2N_2	I_3N_3	I_4N_4	I_5N_5
I_1N_4	I_2N_5	I_3N_1	I_4N_2	I_5N_3	I_1N_3	I_2N_4	I_3N_5	I_4N_1	I_5N_2
I_1A	N_1A	I_2A	N_2A	I_3A	N_3A	I_4A	N_4A	I_5A	N_5A
I_1B	N_1B	I_2B	N_2B	I_3B	N_3B	I_4B	N_4B	I_5B	N_5B
I_1C	N_1C	I_2C	N_2C	I_3C	N_3C	I_4C	N_4C	I_5C	N_5C
I_1D	N_1D	I_2D	N_2D	I_3D	N_3D	I_4D	N_4D	I_5D	N_5D
I_1	N_1	I_2	N_2	I_3	N_3	I_4	N_4	I_5	N_5

I_1N_1	I_2N_2	I_3N_3	I_4N_4	I_5N_5	I_1N_4	I_2N_5	I_3N_1	I_4N_2	I_5N_3
I_1N_3	I_2N_4	I_3N_5	I_4N_1	I_5N_2	I_1N_1	I_2N_2	I_3N_3	I_4N_4	I_5N_5
I_1A	N_1A	I_2A	N_2A	I_3A	N_3A	I_4A	N_4A	I_5A	N_5A
I_1B	N_1B	I_2B	N_2B	I_3B	N_3B	I_4B	N_4B	I_5B	N_5B
I_1C	N_1C	I_2C	N_2C	I_3C	N_3C	I_4C	N_4C	I_5C	N_5C
I_1D	N_1D	I_2D	N_2D	I_3D	N_3D	I_4D	N_4D	I_5D	N_5D
C	D	A	B	C	D	A	B	C	D
I_1	N_1	I_2	N_2	I_3	N_3	I_4	N_4	I_5	N_5
$I_2\ I_2\ I_2\ I_2\ I_2\ I_2\ I_2$<br>$I_2\ I_2\ I_2\ I_2\ I_2\ I_2\ I_2$<br>$I_2\ I_2\ I_2\ I_2\ I_2\ I_2\ I_2$<br>$I_2\ I_2\ I_2\ I_2\ I_2\ I_2\ I_2$<br>$I_2\ I_2\ I_2\ I_2\ I_2\ I_2\ I_2$<br>$I_2\ I_2\ I_2\ I_2\ I_2\ I_2\ I_2$<br>$I_2\ I_2\ I_2\ I_2\ I_2\ I_2\ I_2$				$N_1\ N_1\ N_1\ N_1\ N_1\ N_1\ N_1$<br>$N_1\ N_1\ N_1\ N_1\ N_1\ N_1\ N_1$<br>$N_1\ N_1\ N_1\ N_1\ N_1\ N_1\ N_1$<br>$N_1\ N_1\ N_1\ N_1\ N_1\ N_1\ N_1$<br>$N_1\ N_1\ N_1\ N_1\ N_1\ N_1\ N_1$<br>$N_1\ N_1\ N_1\ N_1\ N_1\ N_1\ N_1$<br>$N_1\ N_1\ N_1\ N_1\ N_1\ N_1\ N_1$				$I_3\ I_3\ I_3\ I_3\ I_3\ I_3\ I_3$<br>$I_3\ I_3\ I_3\ I_3\ I_3\ I_3\ I_3$<br>$I_3\ I_3\ I_3\ I_3\ I_3\ I_3\ I_3$<br>$I_3\ I_3\ I_3\ I_3\ I_3\ I_3\ I_3$<br>$I_3\ I_3\ I_3\ I_3\ I_3\ I_3\ I_3$<br>$I_3\ I_3\ I_3\ I_3\ I_3\ I_3\ I_3$<br>$I_3\ I_3\ I_3\ I_3\ I_3\ I_3\ I_3$	
I_1N_4	I_2N_5	I_3N_1	I_4N_2	I_5N_3	I_1N_3	I_2N_4	I_3N_5	I_4N_1	I_5N_2
I_1N_1	I_2N_2	I_3N_3	I_4N_4	I_5N_5	I_1N_4	I_2N_5	I_3N_1	I_4N_2	I_5N_3
I_1A	N_1A	I_2A	N_2A	I_3A	N_3A	I_4A	N_4A	I_5A	N_5A
I_1B	N_1B	I_2B	N_2B	I_3B	N_3B	I_4B	N_4B	I_5B	N_5B
I_1C	N_1C	I_2C	N_2C	I_3C	N_3C	I_4C	N_4C	I_5C	N_5C
I_1D	N_1D	I_2D	N_2D	I_3D	N_3D	I_4D	N_4D	I_5D	N_5D
I_1	N_1	I_2	N_2	I_3	N_3	I_4	N_4	I_5	N_5
$N_3\ N_3\ N_3\ N_3\ N_3\ N_3\ N_3$<br>$N_3\ N_3\ N_3\ N_3\ N_3\ N_3\ N_3$<br>$N_3\ N_3\ N_3\ N_3\ N_3\ N_3\ N_3$<br>$N_3\ N_3\ N_3\ N_3\ N_3\ N_3\ N_3$<br>$N_3\ N_3\ N_3\ N_3\ N_3\ N_3\ N_3$<br>$N_3\ N_3\ N_3\ N_3\ N_3\ N_3\ N_3$<br>$N_3\ N_3\ N_3\ N_3\ N_3\ N_3\ N_3$				$I_4\ I_4\ I_4\ I_4\ I_4\ I_4\ I_4$<br>$I_4\ I_4\ I_4\ I_4\ I_4\ I_4\ I_4$<br>$I_4\ I_4\ I_4\ I_4\ I_4\ I_4\ I_4$<br>$I_4\ I_4\ I_4\ I_4\ I_4\ I_4\ I_4$<br>$I_4\ I_4\ I_4\ I_4\ I_4\ I_4\ I_4$<br>$I_4\ I_4\ I_4\ I_4\ I_4\ I_4\ I_4$<br>$I_4\ I_4\ I_4\ I_4\ I_4\ I_4\ I_4$				$N_5\ N_5\ N_5\ N_5\ N_5\ N_5\ N_5$<br>$N_5\ N_5\ N_5\ N_5\ N_5\ N_5\ N_5$<br>$N_5\ N_5\ N_5\ N_5\ N_5\ N_5\ N_5$<br>$N_5\ N_5\ N_5\ N_5\ N_5\ N_5\ N_5$<br>$N_5\ N_5\ N_5\ N_5\ N_5\ N_5\ N_5$<br>$N_5\ N_5\ N_5\ N_5\ N_5\ N_5\ N_5$<br>$N_5\ N_5\ N_5\ N_5\ N_5\ N_5\ N_5$	
I_1N_3	I_2N_4	I_3N_5	I_4N_1	I_5N_2	I_1N_1	I_2N_2	I_3N_3	I_4N_4	I_5N_5
I_1A	N_1A	I_2A	N_2A	I_3A	N_3A	I_4A	N_4A	I_5A	N_5A
I_1B	N_1B	I_2B	N_2B	I_3B	N_3B	I_4B	N_4B	I_5B	N_5B
I_1C	N_1C	I_2C	N_2C	I_3C	N_3C	I_4C	N_4C	I_5C	N_5C
I_1D	N_1D	I_2D	N_2D	I_3D	N_3D	I_4D	N_4D	I_5D	N_5D
A	B	C	D	A	B	C	D	A	B
I_1	N_1	I_2	N_2	I_3	N_3	I_4	N_4	I_5	N_5
I_1N_4	I_2N_5	I_3N_1	I_4N_2	I_5N_3	I_1N_3	I_2N_4	I_3N_5	I_4N_1	I_5N_2
I_1A	N_1A	I_2A	N_2A	I_3A	N_3A	I_4A	N_4A	I_5A	N_5A

I_1B	N_1B	I_2B	N_2B	I_3B	N_3B	I_4B	N_4B	I_5B	N_5B
I_1C	N_1C	I_2C	N_2C	I_3C	N_3C	I_4C	N_4C	I_5C	N_5C
I_1D	N_1D	I_2D	N_2D	I_3D	N_3D	I_4D	N_4D	I_5D	N_5D
C	D	A	B	C	D	A	B	C	D
I_1	N_1	I_2	N_2	I_3	N_3	I_4	N_4	I_5	N_5
I_1N_1	I_2N_2	I_3N_3	I_4N_4	I_5N_5	I_1N_4	I_2N_5	I_3N_1	I_4N_2	I_5N_3
I_1A	N_1A	I_2A	N_2A	I_3A	N_3A	I_4A	N_4A	I_5A	N_5A
I_1B	N_1B	I_2B	N_2B	I_3B	N_3B	I_4B	N_4B	I_5B	N_5B
I_2 I_2 I_2 I_2 I_2 I_2 I_2<br>I_2 I_2 I_2 I_2 I_2 I_2 I_2<br>I_2 I_2 I_2 I_2 I_2 I_2 I_2<br>I_2 I_2 I_2 I_2 I_2 I_2 I_2<br>I_2 I_2 I_2 I_2 I_2 I_2 I_2<br>I_2 I_2 I_2 I_2 I_2 I_2 I_2<br>I_2 I_2 I_2 I_2 I_2 I_2 I_2				N_1 N_1 N_1 N_1 N_1 N_1 N_1<br>N_1 N_1 N_1 N_1 N_1 N_1 N_1<br>N_1 N_1 N_1 N_1 N_1 N_1 N_1<br>N_1 N_1 N_1 N_1 N_1 N_1 N_1<br>N_1 N_1 N_1 N_1 N_1 N_1 N_1<br>N_1 N_1 N_1 N_1 N_1 N_1 N_1<br>N_1 N_1 N_1 N_1 N_1 N_1 N_1				I_3 I_3 I_3 I_3 I_3 I_3 I_3<br>I_3 I_3 I_3 I_3 I_3 I_3 I_3<br>I_3 I_3 I_3 I_3 I_3 I_3 I_3<br>I_3 I_3 I_3 I_3 I_3 I_3 I_3<br>I_3 I_3 I_3 I_3 I_3 I_3 I_3<br>I_3 I_3 I_3 I_3 I_3 I_3 I_3<br>I_3 I_3 I_3 I_3 I_3 I_3 I_3	
I_1C	N_1C	I_2C	N_2C	I_3C	N_3C	I_4C	N_4C	I_5C	N_5C
I_1D	N_1D	I_2D	N_2D	I_3D	N_3D	I_4D	N_4D	I_5D	N_5D
I_1	N_1	I_2	N_2	I_3	N_3	I_4	N_4	I_5	N_5
I_1N_3	I_2N_4	I_3N_5	I_4N_1	I_5N_2	I_1N_1	I_2N_2	I_3N_3	I_4N_4	I_5N_5
I_1A	N_1A	I_2A	N_2A	I_3A	N_3A	I_4A	N_4A	I_5A	N_5A
I_1B	N_1B	I_2B	N_2B	I_3B	N_3B	I_4B	N_4B	I_5B	N_5B
I_1C	N_1C	I_2C	N_2C	I_3C	N_3C	I_4C	N_4C	I_5C	N_5C
N_3 N_3 N_3 N_3 N_3 N_3 N_3<br>N_3 N_3 N_3 N_3 N_3 N_3 N_3<br>N_3 N_3 N_3 N_3 N_3 N_3 N_3<br>N_3 N_3 N_3 N_3 N_3 N_3 N_3<br>N_3 N_3 N_3 N_3 N_3 N_3 N_3<br>N_3 N_3 N_3 N_3 N_3 N_3 N_3<br>N_3 N_3 N_3 N_3 N_3 N_3 N_3				I_4 I_4 I_4 I_4 I_4 I_4 I_4<br>I_4 I_4 I_4 I_4 I_4 I_4 I_4<br>I_4 I_4 I_4 I_4 I_4 I_4 I_4<br>I_4 I_4 I_4 I_4 I_4 I_4 I_4<br>I_4 I_4 I_4 I_4 I_4 I_4 I_4<br>I_4 I_4 I_4 I_4 I_4 I_4 I_4<br>I_4 I_4 I_4 I_4 I_4 I_4 I_4				N_5 N_5 N_5 N_5 N_5 N_5 N_5<br>N_5 N_5 N_5 N_5 N_5 N_5 N_5<br>N_5 N_5 N_5 N_5 N_5 N_5 N_5<br>N_5 N_5 N_5 N_5 N_5 N_5 N_5<br>N_5 N_5 N_5 N_5 N_5 N_5 N_5<br>N_5 N_5 N_5 N_5 N_5 N_5 N_5<br>N_5 N_5 N_5 N_5 N_5 N_5 N_5	
I_1D	N_1D	I_2D	N_2D	I_3D	N_3D	I_4D	N_4D	I_5D	N_5D
I_1	N_1	I_2	N_2	I_3	N_3	I_4	N_4	I_5	N_5
I_1N_4	I_2N_5	I_3N_1	I_4N_2	I_5N_3	I_1N_3	I_2N_4	I_3N_5	I_4N_1	I_5N_2
I_1A	N_1A	I_2A	N_2A	I_3A	N_3A	I_4A	N_4A	I_5A	N_5A
I_1B	N_1B	I_2B	N_2B	I_3B	N_3B	I_4B	N_4B	I_5B	N_5B
I_1C	N_1C	I_2C	N_2C	I_3C	N_3C	I_4C	N_4C	I_5C	N_5C
I_1D	N_1D	I_2D	N_2D	I_3D	N_3D	I_4D	N_4D	I_5D	N_5D
